# Burden and challenges in managing TB infection among people with occupational exposure to silica in India

**DOI:** 10.5588/ijtldopen.24.0402

**Published:** 2024-11-01

**Authors:** B. Kalottee, P. Mahajan, A. Nuken, D. Nair, P. Thekkur, A.M.V. Kumar, V. Rai, M. Parmar, H. Solanki, R. Rao, S.K. Mattoo, R. Kumar

**Affiliations:** ^1^ International Union Against Tuberculosis and Lung Disease (The Union), South-East Asia Office, New Delhi, India;; ^2^ Centre for Operational Research, The Union, Paris, France;; ^3^ Directorate of Health Services, Madhya Pradesh, Bhopal, India;; ^4^ World Health Organization (WHO), Country Office for India, New Delhi, India;; ^5^ Central TB Division, Directorate General of Health Services, Ministry of Health & Family Welfare, New Delhi, India;; ^6^ RHTC Hospital, Ministry of Health & Family Welfare, Najafgarh, New Delhi, India.

**Keywords:** tuberculosis, mining, occupational health, TB preventive treatment, TPT

## Abstract

**BACKGROUND:**

Occupational exposure to silica increases the risk of TB infection (TBI) and disease. This study aimed to determine the prevalence of TBI and explore challenges in TBI management in such individuals in two districts of India during 2023.

**METHODS:**

This was an explanatory mixed-methods study with a quantitative cohort design and qualitative descriptive in-depth interviews.

**RESULTS:**

Among 1,555 individuals with occupational exposure to silica, 593 (38%, 95% CI 36–41) underwent interferon-gamma release assay (IGRA) for TBI, of whom 255 (43%, 95% CI 39–47) were found IGRA-positive. Males with occupational silica exposure for ≥20 years had a significantly higher risk of TBI. Of these 160 individuals eligible for TB preventive therapy (TPT), 153 (96%, 95% CI 92–98) were initiated on TPT and 124 (81%, 95% CI 74–88) completed TPT. The low uptake of IGRA was attributed to the stigma associated with TB and reluctance to undergo any medical evaluation.

**CONCLUSIONS:**

Compared to the general population, individuals with occupational exposure to silica have an almost two times higher prevalence of TBI. Further research is required to identify the threshold of silica exposure to be considered for screening for TBI. Efforts to increase awareness and decrease stigma can improve the uptake of testing for TBI and TB disease.

## RÉSUMÉ

**CONTEXTE:**

L'exposition professionnelle à la silice augmente le risque d'infection tuberculeuse (TBI) et de maladie. Cette étude visait à déterminer la prévalence des traumatismes crâniens et à explorer les défis de la gestion des traumatismes crâniens chez ces personnes dans deux districts de l'Inde en 2023.

**MÉTHODES:**

Il s'agissait d'une étude explicative à méthodes mixtes avec un plan de cohorte quantitatif et des entretiens qualitatifs descriptifs approfondis.

**RÉSULTATS:**

Parmi 1 555 personnes exposées professionnellement à la silice, 593 (38%, IC à 95% 36–41) ont subi un test de libération d'interféron gamma (IGRA, pour l’anglais “interferon-gamma release assay) pour un TBI, dont 255 (43%, IC à 95% 39–47) ont été trouvés positifs au IGRA. Les hommes ayant été exposés à la silice pendant ≥ 20 ans présentaient un risque significativement plus élevé de traumatisme crânien. De ces 160 personnes éligibles au traitement préventif de la TB (TPT), 153 (96%, IC à 95% 92–98) ont commencé à recevoir un TPT et 124 (81%, IC à 95% 74–88) ont terminé le TPT. La faible utilisation des IGRA a été attribuée à la stigmatisation associée à la TB et à la réticence à se soumettre à une évaluation médicale.

**CONCLUSIONS:**

Par rapport à la population générale, les personnes exposées professionnellement à la silice ont une prévalence presque deux fois plus élevée de TBI. D'autres recherches sont nécessaires pour déterminer le seuil d'exposition à la silice à prendre en compte pour le dépistage des TBI. Les efforts visant à accroître la sensibilisation et à réduire la stigmatisation peuvent améliorer l'adoption des tests de dépistage des TBI et de la TB.

The WHO recommends early detection and treatment of all forms of TB disease and scaling-up of detection of TB infection (TBI) and TB preventive treatment (TPT) in high-risk groups.^1^ These groups are identified based on their elevated risk of progression from TBI to TB disease or increased likelihood of exposure to TBI. Among these high-risk groups are people diagnosed with silicosis,^2^ as exposure to silica, even without established disease, is associated with an increased risk of TBI and TB disease.^3–8^ The latency period for TBI or TB disease after silica exposure can vary. One study indicated an average latency period of 7.6 years, which can range up to 20 years regardless of the level/intensity of silica dust exposure.^9^ Mining and associated activities expose workers to high levels of silica dust, and exposure to respirable crystalline silica (silica dust particles <4 μm) is associated with pulmonary infections.^10^ In India, an estimated 11.5 million people are employed in occupations exposed to silica dust and are at high risk of TBI.^11^ It is therefore important to identify TBI and ensure timely initiation of TPT to curtail future transmission, morbidity and mortality due to TB.

Because of limited resources for the detection of TBI and providing TPT, many low- and middle-income countries have focused on treating TB disease.^12,13^ However, the commitment of the WHO End TB Strategy and United Nations High-Level Meeting (UNHLM) to TPT has led to a shift in focus to identifying and treating TBI.^1,14^ The National TB Elimination Programme (NTEP) in India considers those with silicosis as a high-risk group for TBI and risk of progression to TB disease.^13^ However, the program currently prioritises household contacts and people living with HIV. Despite the substantial number of people exposed to silica dust, information on the prevalence of TBI is limited. Furthermore, there is no information on the challenges of providing TPT for those with TBI in these risk groups. The present study aimed to estimate the prevalence of TBI and the TPT initiation and completion rates among individuals with occupational exposure to silica dust. The study also aimed to document the facilitators and barriers to TBI management.

## METHODS

### Study design

This exploratory mixed-methods study integrates quantitative and qualitative data collection and analysis. The quantitative component used a cohort design, and the qualitative component used a descriptive design.

### Setting

The study was conducted in Satna and Mandsaur Districts of Madhya Pradesh (a central state of India) and was based on silica dust-producing industries such as mining and quarrying. The Axshya Plus is a large-scale project implemented by The Union, India Office of the International Union Against Tuberculosis and Lung Disease (The Union) in 108 priority districts of seven states, including Madhya Pradesh. Funding support was provided by The Global Fund (2021–2024) for the programmatic implementation of TPT among household contacts of TB patients.^15^ The total population of Mandsaur was 1,340,411, and that of Satna was 2,228,935.^16^ In Mandsaur, slate pencil manufacturing is the most common source of silica dust (e.g., Binota shale stone has a silica dust content of between 35% and 56%).^17^ In Satna, cement manufacturing is the primary source of silica exposure. The study was conducted between February 2023 and January 2024

### Study population and sample size

After obtaining consent, adults (≥18 years) who worked in the silica exposure industries for one or more years were included in the study. As there were no clear cut-offs on the duration of exposure to silica and risk of TBI or TB disease, all those who had exposure of at least one year at any time during their lifetime were included. The sample size was calculated based on the assumption that the proportion completing TPT among those initiated is 80%, relative precision of 10%, design effect of two for using districts as primary sampling units and 95% confidence limit, the minimum number of individuals initiated on TPT required was 200. To initiate 200 individuals on TPT at a 70% TPT initiation rate, 50% TBI prevalence and eligibility, and 10% TB disease point prevalence, we needed to include 636 individuals exposed to silica dust. Assuming a net loss of 40% at various stages, we aimed to enrol 954 individuals exposed to silica dust in the study.

In-depth interviews (IDIs) were conducted with 13 study participants. Two District Research Coordinators (DRCs) who implemented the study were also interviewed to understand the challenges from a provider perspective. Criterion-based purposive sampling was used to select one participant who refused to undergo the interferon-gamma release assay (IGRA) test, one who refused to take TPT, two who were initiated on but did not complete TPT and nine who completed TPT.

### Study procedure

A DRC was appointed in each district and underwent a 2-day training on the study protocol. They collected the study data on a paper-based structured questionnaire at different time points during the study period.

1. Identification of eligible study population: The villages in the vicinity (under <15 km) of the industries associated with silica exposure were mapped through house-to-house visits. Those who had worked or were currently working in the silica-exposure industries for one or more years were line-listed.
2. Enrolment: The study team attempted to meet all line-listed individuals and enrolled them after obtaining informed consent. Individuals were sequentially enrolled till all the line-listed individuals in a village were contacted, even if the required sample size was exceeded.
3. Symptom screening: The study team screened all enrolled participants for four symptoms of TB (i.e., cough for >2 weeks, fever, weight loss, and night sweats). Those with symptoms were referred to their nearest health facility for evaluation for TB disease as per NTEP guidelines through a sputum test or cartridge-based nucleic-acid amplification test (CBNAAT). If they were not found TB active, they were tested with IGRA for TPT.
4. IGRA testing: A trained phlebotomist collected blood samples from asymptomatic individuals at their homes. Those found positive on IGRA (QuantiFERON-TB^®^ Gold In-Tube test; Qiagen, Hilden, Germany) were referred to rule out TB disease with chest X-ray (CXR).
5. Initiation of TPT: In individuals with TBI but no TB disease, the study team arranged for a consultation with the medical officer (MO) in the public healthcare facility who decided the provision of TPT. The TPT regimen in the study districts was 6 months of daily isoniazid (6H), provided free of cost under the NTEP and dispensed through NTEP-affiliated health facilities. The DRC facilitated the delivery of TPT refills to the participants’ homes.
6. Treatment outcome: The DRCs actively followed up with participants initiated on TPT over phone calls or home visits to provide adherence support, check missed doses of medicine refills and facilitate medical consultations to manage adverse events when required. At the end of a course of TPT, the DRCs made home visits to ascertain treatment outcomes. The following outcomes were reported as per NTEP guidelines: treatment completed, treatment discontinued, treatment failed, died, loss to follow-up.^13^ Interview guides containing questions and probes were prepared to elicit information on participants’ perceptions of TBI management. The IDIs were conducted by the DRCs, trained in qualitative interview techniques. Interviews of DRCs were conducted by investigators (AN and PM). All the interviews were conducted in Hindi, the local language, and were audio-recorded with the participants’ consent.

### Data entry and analysis

*Quantitative component:* The DRCs entered the data recorded in the paper-based forms weekly into Epicollect5 (Centre for Genomic Pathogen Surveillance: https://five.epicollect.net). Data were downloaded in an MS Excel (Microsoft, Seattle, WA, USA) format and analysed using STATA^®^ v16.0 (StataCorp, College Station, TX, USA). TPT eligibility, TPT initiation, and TPT outcomes were summarised as proportions with 95% confidence intervals (CIs). Independent factors associated with TBI were identified using adjusted modified Poisson regression among those who underwent IGRA. Adjusted relative risks (aRRs) with 95% CIs were used to measure association.

*Qualitative component:* Hindi transcripts of all the interviews were prepared from the audio recording on the same day of the interview by the DRCs, which were translated into English by PM for further analysis. Two study investigators (PT and DN) trained in qualitative techniques conducted a thematic analysis of the transcripts independently to identify emerging patterns or themes in the qualitative data. Any differences between the investigators were resolved in consultation with a third investigator (AN). The conduct and reporting of the quantitative and qualitative components of the study followed STROBE and COREQ guidelines, respectively.^18,19^

The study was approved by the Ethics Advisory Group, International Union Against Tuberculosis and Lung Disease, Paris, France (EAG No. 06/2022 dated 08/09/2022) and from the Sigma Institutional Review Board, India (IRB No. 10061/IRB/22-23 dated 05/11/2022). Informed consent was obtained from all study participants before enrolment.

## RESULTS

The cascade of events from enrolment to TPT completion is shown in Supplementary Figure S1. In total, 1,555 individuals were listed as exposed to silica dust at their workplace. Of these, the mean age was 41 years (standard deviation [SD] 12); 345 (22%) were females. Of the 1,555, only four were reported to have symptoms suggestive of TB, and one was eventually diagnosed with TB. Of the 1,554 eligible for the IGRA test, 593 (38%, 95% CI 36–41) underwent the IGRA test, of whom 255 (43%, 95% CI 39–47) were found IGRA-positive. Of the 255 IGRA-positive individuals, 178 (70%, 95% CI 64–75) underwent CXR, of whom 174 (98%) had CXR, which was not suggestive of TB. Of the 174 individuals with TBI but not TB disease, 160 (92%, 95% CI 87–95) were eligible for TPT, and 153 (96%, 95% CI 92–98) of the eligible were initiated on TPT. Of the 153 individuals initiated on TPT, 124 (81%, 95% CI 74–88) completed 6 months of treatment.

### Line-listing and screening for TB symptoms

Community engagement and multiple home visits by DRCs facilitated line listing and screening for participants with TB symptoms. It was a challenging process as individuals were sparsely spread out in the districts, and people were reluctant to undergo screening and disclose symptoms for fear of losing their jobs and the stigma associated with a diagnosis of silicosis or TB. Thus, only four individuals reported symptoms suggestive of TB disease.

### Testing for TBI

Only 38% of eligible individuals underwent an IGRA test (Table 1). Uptake of IGRA test was low among males (35%), those with current occupational exposure to silica (37%) and those in the age group of 31 to 45 years (35%). Higher IGRA positivity was seen in males (46%), those aged ≥61 years (47%), those with current occupational exposure to silica (44%), occupational exposure for ≥20 years (53%) and participants from Mandsaur (48%). Males (aRR 1.3, 95% CI 1.0–1.6) and those with occupational silica exposure for ≥20 years (aRR 1.6; 95% CI 1.1–2.3) had significantly higher risk of TBI (Supplementary Table S1). Major facilitators were the availability of IGRA testing free of cost and doorstep sample collection. Despite efforts by the DRCs to counsel and raise awareness of TBI and TPT during line-listing/home visits, major barriers were the fear of diagnosis of TB, apprehension of family members for the IGRA test and non-availability of eligible individuals during visits for sample collection (Figure).

**Table 1.** IGRA and CXR uptake, and IGRA positivity among those with occupational exposure to silica, Satna and Mandsaur Districts, India.

Characteristics	Total	Underwent IGRA	Positive on IGRA	Underwent chest X-ray
	*n*	*n* (%)*	*n* (%)^†^	*n* (%)^‡^
Total	1,555	593 (38.1)	255 (43.0)	178 (69.8)
Age group, years				
18–30	384	147 (38.3)	53 (36.1)	25 (47.2)
31–45	631	219 (34.7)	97 (44.3)	71 (73.2)
46–60	452	176 (38.9)	81 (46.0)	65 (80.2)
≥61	88	51 (58.0)	24 (47.1)	17 (70.8)
Sex				
Male	1,210	417 (34.5)	190 (45.6)	135 (71.1)
Female	345	176 (51.0)	65 (36.9)	43 (66.2)
Type of exposure				
Direct^§^	1,122	443 (39.5)	191 (43.1)	130 (68.1)
Indirect^¶^	433	150 (34.6)	64 (42.7)	48 (75.0)
Current exposure				
No	206	98 (47.6)	36 (36.7)	27 (75.0)
Yes	1,349	495 (36.7)	219 (44.2)	151 (68.9)
Duration of exposure, years				
1–5	405	146 (36.0)	47 (34.2)	26 (43.8)
6–10	419	142 (33.9)	57 (40.1)	40 (70.2)
11–19	331	120 (36.3)	53 (44.2)	42 (79.2)
≥20	400	185 (46.3)	98 (53.0)	70 (71.4)
District				
Mandsaur	584	241 (41.3)	115 (47.7)	76 (66.1)
Satna	971	352 (36.3)	140 (39.8)	102 (72.9)

* Row percentage was calculated using line-listed individuals with occupational silica exposure as the denominator.

† Row percentage was calculated using the number of those who underwent IGRA testing as the denominator.

‡ Row percentage was calculated using the number of individuals who underwent positive IGRA testing as the denominator.

§ Occurs when someone is near the source of silica dust generation and is actively inhaling dust particles (e.g., mining and quarrying.).

¶ Occurs when an individual is not directly engaged in activities that involve the active inhalation of dust particles (e.g., support staff).

IGRA = interferon-gamma release assay; CXR = chest X-ray.

**Figure.**
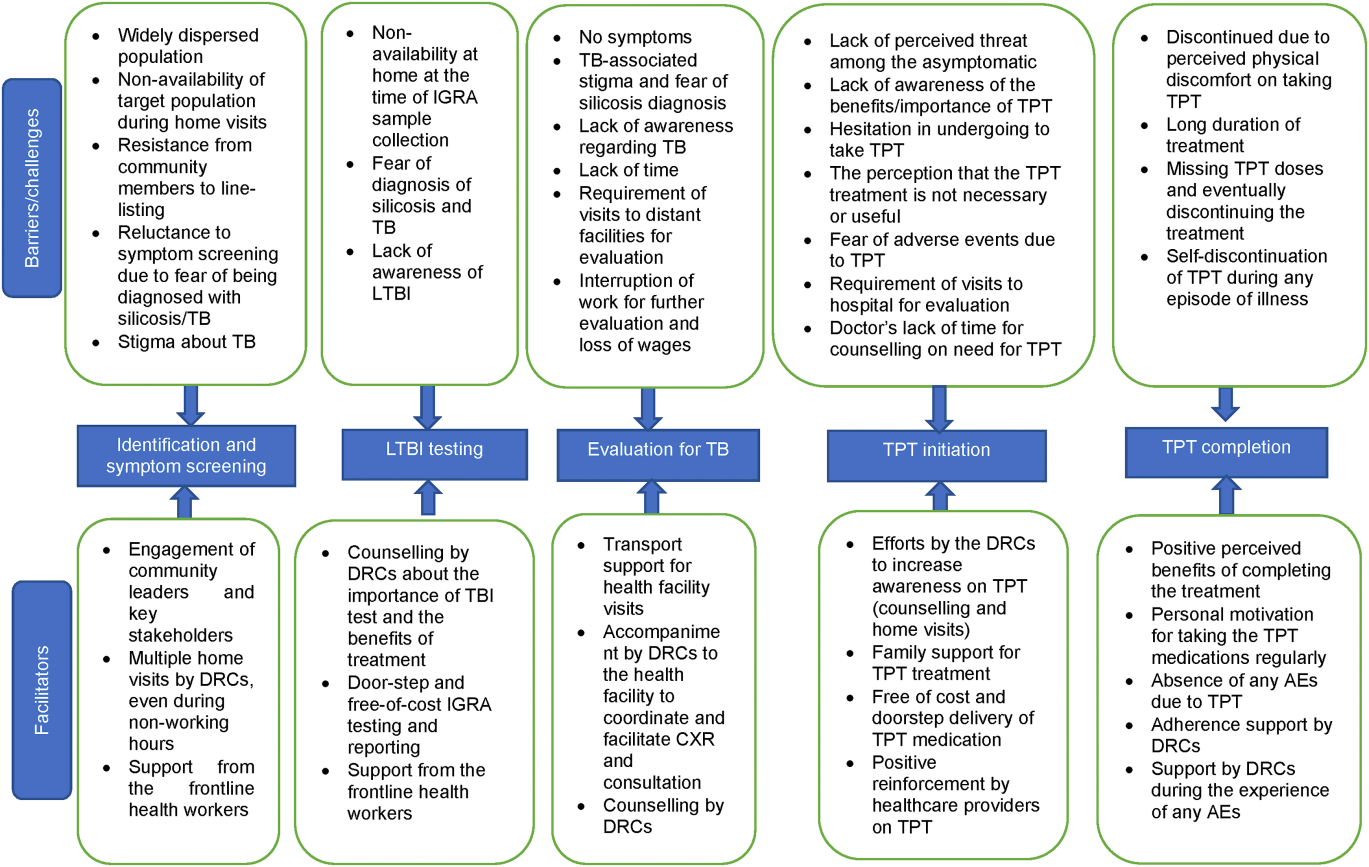
Barriers and facilitators in the management of TB infection among those with occupational exposure to silica in Satna and Mandsaur Districts of Madhya Pradesh, India. IGRA = interferon-gamma release assay; LTBI = latent TB infection; TPT = TB prevention therapy; DRC = District Research Coordinator; CXR = chest X-ray.

### Evaluation for TB disease

The proportion of participants with TBI who underwent CXR to rule out TB disease was lower among those aged 18–30 years (47%), female (66%), those with occuaptional exposure of up to 5 years (44%) and partcipants from Mandsaur (66%) (Table 1). (*This should be Table 1. Please confirm*) This was mainly because they were asymptomatic and did not feel the need for further evaluation, the stigma around TB and fear of losing their job if diagnosed with TB, distant health facilities, interruption of work and loss of wages due to facility visits. Facilitators were cited as support for transport costs and DRCs motivating and accompanying them to health facilities (Figure).

**Table 2.** TPT eligibility, initiation, and completion among those with occupational exposure to silica, Satna and Mandsaur Districts, India.

Characteristics	Total	Eligible for TPT	Initiated on TPT	Completed TPT
	*n*	*n* (%)*	*n* (%)^†^	*n* (%)^‡^
Total	174	160 (92.0)	153 (95.6)	124 (81.1)
Age group, years				
18–30	25	25 (100.0)	22 (88.0)	20 (90.9)
31–45	71	67 (94.4)	64 (95.5)	52 (81.3)
46–60	62	53 (85.5)	52 (98.1)	42 (80.8)
≥61	16	15 (93.8)	15 (100.0)	10 (66.7)
Sex				
Male	133	124 (93.2)	119 (96.0)	101 (84.9)
Female	41	36 (87.8)	34 (94.4)	23 (67.6)
Type of exposure				
Direct^§^	126	115 (91.3)	108 (93.9)	85 (78.7)
Indirect^¶^	48	45 (93.8)	45 (100.0)	39 (86.7)
Current exposure				
No	26	20 (76.9)	20 (100.0)	13 (65.0)
Yes	148	140 (94.6)	133 (95.0)	111 (83.5)
Duration of exposure, years				
1–5	26	26 (100.0)	26 (100.0)	7 (100.0)
6–10	40	39 (97.5)	36 (92.3)	12 (63.2)
11–19	41	39 (95.1)	36 (92.3)	30 (83.3)
≥20	67	56 (83.6)	55 (98.2)	33 (91.7)
District				
Mandsaur	72	65 (90.3)	59 (90.8)	43 (72.9)
Satna	102	160 (92.0)	94 (98.9)	81 (86.2)

* Row percentage calculated using individuals eligible for TPT based on IGRA-positive and those with normal chest X-ray result as denominator.

† Row percentage calculated with those eligible for TPT after assessment by medical officer as the denominator.

‡ Row percentage calculated with those initiated on TPT as the denominator.

§ Occurs when someone is near the source of silica dust generation and is actively inhaling dust particles (e.g., mining and quarrying).

¶ Occurs when someone is not directly involved in activities that directly expose to active inhaling of dust particles (e.g., support staff).

TPT = TB prevention therapy; IGRA = interferon-gamma release assay.

### Initiation of TPT

Among those eligible for TPT, close to 90% were initiated on TPT across all age groups, genders, and occupational exposure categories (Table 2). TPT uptake was facilitated by medical doctors and health staff counselling on the importance of TPT initiation and doorstep delivery of TPT medicines by the DRCs (and their efforts to advise and motivate the participants and their family members to continue treatment). Hesitancy to take TPT in asymptomatic and healthy participants and fear of adverse events prevented some from taking TPT (Figure).

### Completion of TPT

Among those initiated on TPT, completion was higher among those aged 18–30 years (91%) (Table 2). During IDIs, long duration of treatment self-discontinuation due to discomfort were cited as reasons for interrupting or discontinuing TPT. Positive re-enforcement by the DRCs motivated participants to complete the course of TPT (Figure).

## DISCUSSION

We report on the cascade of TBI management among those with occupational exposure to silica dust in India. The study has three key findings: 1) higher prevalence of TBI among those with occupational exposure to silica compared to the general population, 2) high uptake and completion of TPT and 3) low rate of presumptive TB detection, low IGRA and CXR uptake mainly due to the stigma and fear of losing their jobs if diagnosed with TB.

The study has a few strengths. The community-based sampling from two districts where people engaged in the industry’s risk for silicosis provided TBI estimates for people with varied sources of silica exposure. The IDIs were guided by the quantitative findings, and combining the two techniques provided a better understanding of the challenges in TBI management. There were also limitations. Due to high attrition in parts of the cascade of TBI management, regression analysis to identify factors associated with TBI detection, TPT initiation and completion could not be undertaken. Due to operational constraints, evaluation for silicosis could not be conducted, and therefore, we are unable to provide estimates of TBI in those with silicosis. We were also unable to conduct qualitative interviews among programmatic staff and to explore their perspectives on the implementation of TBI management in this study population. Despite these limitations, the study has important implications.

First, the prevalence of TBI among those with occupational exposure to silica was 43%, which is higher than the 23% reported in the general population in India and the global prevalence of 25%.^20^ Risk of TBI was higher in males and those with exposure for ≥20 years. The presence of silica particles in the lung facilitates the initiation of TBI, progression to TB disease and long-standing TB-associated disability through multiple pathological pathways.^9^ The association between long-term silica dust exposure (>20 years) and a higher risk of TBI suggests that some participants may have undiagnosed silicosis. However, this cannot be confirmed without appropriate testing. Though global and national guidelines recommend TPT for those diagnosed with silicosis, the diagnosis of silicosis is challenging in primary care settings. Further research should be conducted to identify thresholds for the extent and duration of silica dust exposure. This could guide policies on identifying high-risk groups to be assessed for TBI.

Second, there was high uptake and completion of TPT among those in whom TB disease was ruled out. This was possible due to the specific training provided to the study team and their focused efforts in counselling participants and their families regarding TPT. The re-enforcement of these messages by healthcare staff also motivated participants to initiate and complete TPT. Studies have highlighted the importance of structured counselling to improve awareness and uptake of TPT in high-risk groups (e.g., household contacts of patients with TB and those with HIV).^21–23^ The programmatic management of TPT should include specific training of healthcare workers on counselling silica-exposed individuals on TPT benefits and adherence support.

Third, the study participants were hesitant to disclose their symptoms and undergo any evaluation for TBI or TB disease due to stigma and fear of losing their jobs. This led to a low rate of presumptive TB detection and low IGRA and CXR uptake. The current occupational health legislation and social protection initiatives must be strictly enforced or strengthened to ensure that a worker’s right to health is upheld without compromising their job security.

A possible intervention among silica-exposed individuals/workers could be to integrate screening during the periodic health checks conducted at the workplace or in the community. This could use symptoms and CXR-based screening, with Cy-tb skin test for TBI and rapid molecular test for TB, followed by appropriate TPT or TB treatment. There is a need to consider strategies for translating TBI management into real-life settings. In the current study, even with dedicated research staff to facilitate the screening process and provide financial support, the uptake of TBI testing was sub-optimal, and of those who started TBI, 20% did not complete the course. This raises concerns about the risk of ongoing transmission and the development of isoniazid resistance due to an incomplete course of TPT. A more comprehensive approach is needed to identify and treat all potential TBI/TB cases on a broader scale. Institutional mechanisms for periodic workplace screening for TBI should be considered. Also, existing healthcare workers, as well as the recently introduced community health volunteers, and TB survivors, could be trained to conduct community-level screenings supported by mobile health units and outreach camps. Community health volunteers and TB survivors who have played an important role in the care provision for TB patients can also be trained to increase awareness and decrease stigma surrounding TB to improve uptake of screening, testing and treatment adherence.

## CONCLUSION

Compared to the general population, individuals with occupational exposure to silica have an almost two times higher prevalence of TBI. However, study participants were hesitant to undergo evaluations for TBI or TB disease due to stigma and fear of losing their jobs. Efforts to increase awareness and decrease stigma can improve the uptake of testing for TBI and TB disease. Health legislation and social protection must also be enforced or strengthened to ensure a worker’s right to health is upheld without compromising job security. Employers and occupational health departments can also play an important role in conducting biannual health screenings, particularly among silica-exposed workers. Addressing these broader health and socioeconomic determinants is crucial in mitigating the risk of TBI and TB disease.

## Supplementary Material


