# Supplementary material for: Burden and challenges in managing TB infection among people with occupational exposure to silica in India
Source: IJTLD Open. 2024 Nov 1;1(11):501–7. doi: 10.5588/ijtldopen.24.0402 (PMC11558788; doi:10.5588/ijtldopen.24.0402)
Supplement: Supplementary file 1 [file ijtldopen24-0402_supplementarydata1.pdf]

http://dx.doi.org/10.5588/ijtldopen.24.0402

Burden and challenges in managing TB infection among people with occupational exposure to silica in India

Supplementary Table S1: Factors associated with TB infection among those with occupational exposure to silica and underwent IGRA testing in Satna and Mandsaur districts of Madhya Pradesh, India

Characteristics	Underwent IGRA	Positive on IGRA	Unadjusted RR		Adjusted RR		
		n (%)	RR	(95 % CI)	RR	(95 % CI)	p-value
**Total**	**593**	**255 (43.0%)**					
Age group (in years)							
18-30	147	53 (36.1%)	Ref		Ref		
31-45	219	97 (44.3%)	1.2	(0.9-1.6)	1.0	(0.7-1.4)	0.985
46-60	176	81 (46.0%)	1.3	(1.0-1.7)	1.0	(0.7-1.4)	0.855
61 and above	51	24 (47.1%)	1.3	(0.9-1.9)	1.1	(0.7-1.8)	0.592
Gender							
Male	417	190 (45.6%)	1.2	(1.0-1.5)	1.3	(1.0-1.6)	0.042
Female	176	65 (36.9%)	Ref		Ref		
Type of exposure							
Direct	443	191 (43.1%)	Ref		Ref		
Indirect	150	64 (42.7%)	1.0	(0.8-1.2)	1.1	(0.8-1.4)	0.703
Current exposure							
No	98	36 (36.7%)	Ref		Ref		
Yes	495	219 (44.2%)	1.2	(0.9-1.6)	1.3	(0.9-1.7)	0.129
Duration of exposure (in years)							
1-5	146	47 (34.2%)	Ref		Ref		
6-10	142	57 (40.1%)	1.2	(0.9-1.7)	1.2	(0.9-1.7)	0.216
11-19	120	53 (44.2%)	1.4	(1.0-1.9)	1.3	(0.9-1.9)	0.100
≥20	185	98 (53.0%)	1.6	(1.3-2.2)	1.6	(1.1-2.3)	0.010

District							
Mandsaur	241	115 (47.7%)	1.2	(1.0-1.4)	1.2	(1.0-1.5)	0.094
Satna	352	140 (39.8%)	Ref		Ref		

Abbreviation: IGRA-Interferon Gamma Release Assay

http://dx.doi.org/10.5588/ijtldopen.24.0402

Supplementary Figure S1. Flowchart depicting the management of TB infection among those with occupational exposure to silica in Satna and Mandsaur Districts of Madhya Pradesh, India.

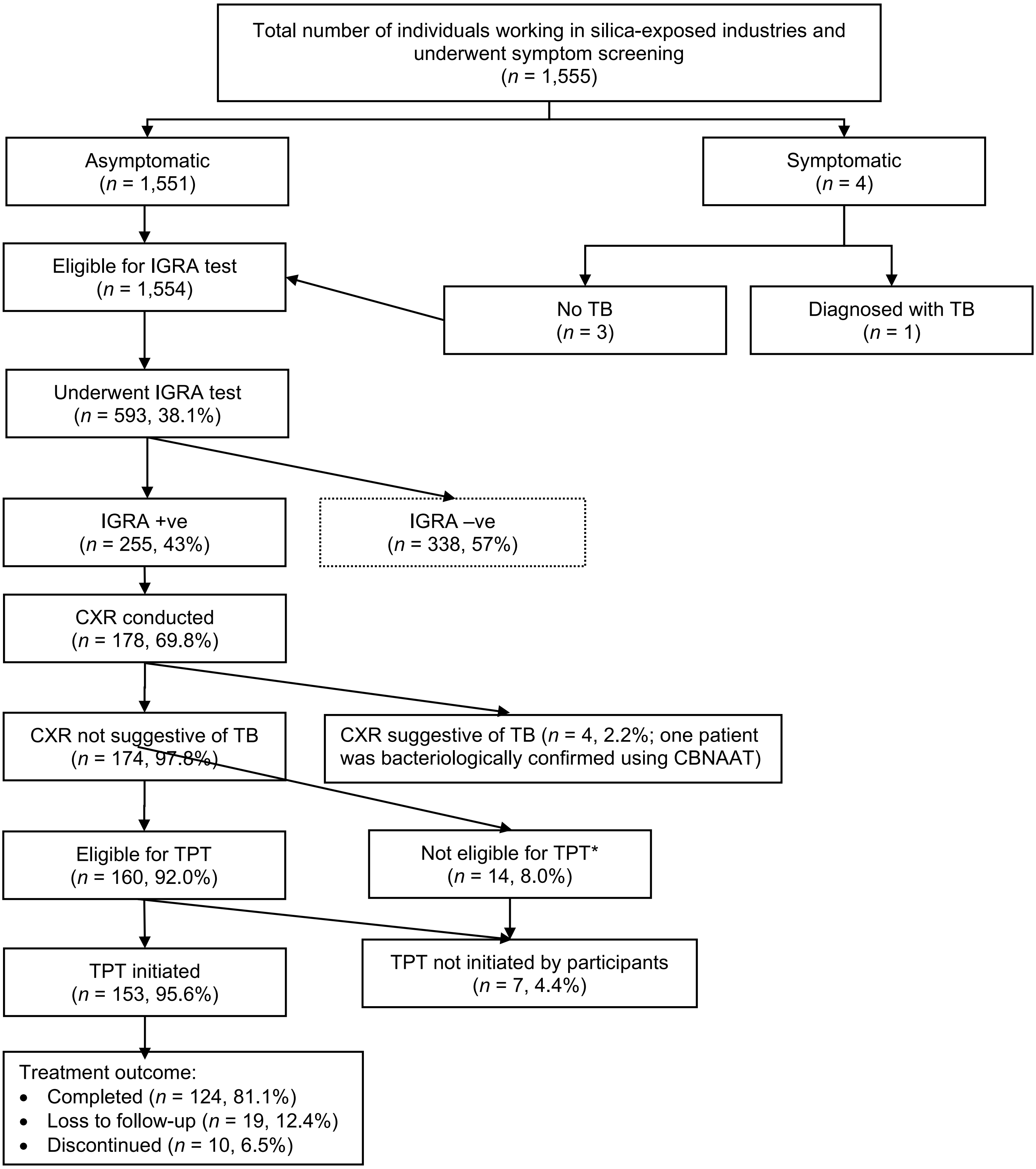

TPT = TB preventive treatment; IGRA = interferon-gamma release assay; CXR = chest X-ray; CBNAAT = cartridge-based nucleic acid amplification test. *MO considered not eligible for TPT due to participant's ongoing treatment such as heart treatment, diabetic and hypertensive.
